# Selection and identification of a novel bone-targeting peptide for biomedical imaging of bone

**DOI:** 10.1038/s41598-020-67522-4

**Published:** 2020-06-29

**Authors:** Jinho Bang, Heesun Park, Jihye Yoo, Donghyun Lee, Won Il Choi, Jin Hyung Lee, Young-Ran Lee, Chungho Kim, Heebeom Koo, Sunghyun Kim

**Affiliations:** 1 Korea Institute of Ceramic Engineering and Technology, Center for Convergence Bioceramic Materials, 202 Osongsaengmyeong 1-ro, Cheongjusi, Chungcheongbuk-do 28160 South Korea; 2 Department of Medical Life Sciences, College of Medicine, The Catholic University of Korea, 222 Banpo-daero, Seocho-gu, Seoul, 06591 South Korea; 3 Department of Life Sciences, Korea University, Seoul, 136-701 South Korea

**Keywords:** Biochemistry, Biotechnology, Chemical biology, Molecular medicine

## Abstract

The global burden of bone-related diseases is increasing in the aging society; thus, improved bone targeted imaging for their early identification and treatment are needed. In this study, we screened novel peptide ligands for hydroxyapatite, a major inorganic component of teeth and bones, and identified a peptide enabling in vivo bone targeting and real-time fluorescence bone detection. To isolate peptides highly specific for hydroxyapatite, we used negative and positive selection from a randomized 8-mer peptide phage library and identified hydroxyapatite-specific peptides (HA-pep2, HA-pep3, and HA-pep7). Among these three peptides, HA-pep3 showed the highest binding capacity and superior dissociation constant towards hydroxyapatite surfaces over time (~ 88.3% retained on hydroxyapatite after two weeks). Furthermore, HA-pep3 was highly specific for hydroxyapatite compared to other calcium salt-based materials. Using this superior specificity, HA-pep3 showed higher accumulation in skull, spine, and joints in comparison with scrambled control peptide during real-time whole-body imaging. Ex vivo analysis of the major organs and bone from mice demonstrated that the fluorescence intensity in bone was about 3.32 folds higher in the case of HA-pep3 than the one exhibited by the scrambled control peptide. Our study identified a novel approach for targeting ligands for bone specific imaging and can be useful for drug delivery applications.

## Introduction

Bone-related diseases are imposing an increasingly heavy burden in the aging society, and they are particularly difficult to treat due to their complex anatomical characteristics. More than 50% of the United States population aged 50 years and older is diagnosed with osteoporosis or low bone mass^1^. Therefore, imaging techniques for the early detection of bone-related disorders are important for their timely identification and treatment. The most widely used bone imaging technologies are X-ray and computed tomography (CT). However, they are potentially harmful due to ionizing radiation exposure, especially when prolonged or frequent imaging is required^2^. Fluorescence imaging can achieve high selectivity and sensitivity, and it is widely used in the biological sciences for both in vitro and in vivo analysis^3,4^. Recently, the development of fluorescence imaging applications for the early detection of bone-related diseases has received increasing attention, because of both advances in the fluorescence microscopy technology and potential risks associated with X-ray and CT imaging^5–7^.

Hydroxyapatite, Ca_5_(PO_4_)_3_(OH), is a polymorph of calcium phosphate (Ca_3_(PO_4_)_2_), in which two hydroxyl groups have been replaced by phosphate groups. Hydroxyapatite, a major inorganic component of teeth and bones, is currently the most commonly clinically used implant material in due to its structure and unique functional properties^8^.

Many targeting ligands for bone or hydroxyapatite imaging have been developed in recent years^9,10^ including phosphonate derivatives or oligopeptides with repeating sequences of acidic amino acids (Asp or Glu). Phosphonate derivatives, and especially bisphosphonates, have been widely applied as bone-targeting ligands in various imaging agents for the diagnosis and therapy of many bone-related diseases, and they exhibit high affinity for hydroxyapatite^11,12^. However, bisphosphonates also have some limitations including poor bioavailability, long half-life in vivo*,* and side effects such as ulcers, osteonecrosis of the jaw, and musculoskeletal pain^13^. Acidic oligopeptides with poly-Glu or poly-Asp amino acids have an affinity to hydroxyapatite in vitro and are selectively targeted into the bone in vivo^14–16^. However, despite intensive research efforts, targeting peptide ligands suitable for in vivo bone imaging are extremely rare, because acidic oligopeptides lack specificity and because ligands only recognize bone matrix with positively charged calcium. Therefore, the development of novel targeting molecules with high selectivity for in vivo bone fluorescence imaging is necessary.

Combinatorial phage display is powerful methodology for the isolation of peptides with high selectivity and binding affinity to both organic and inorganic materials^17–19^. The identification of peptides binding specifically to hydroxyapatite provides opportunities for bone targeting imaging optimization.

In this report, we screened novel peptides with strong binding affinity and specificity for hydroxyapatite from a randomized 8-mer peptide phage library. After isolating hydroxyapatite binding peptide candidates, we investigated the kinetics of their binding to and release from hydroxyapatite, and selected the most effective peptides for bone-targeting fluorescence imaging. Then, we studied the in vitro specificity of HA-targeting peptides to several calcium-based minerals and demonstrated in vivo performance of the peptides in mice after an intravenous injection. To the best of our knowledge, this is the first report describing the isolation of novel hydroxyapatite-binding peptides with high specificity for use in bone-specific imaging.

## Results

### Overview of in vivo bone imaging using the novel peptide ligand

**Figure 1:**
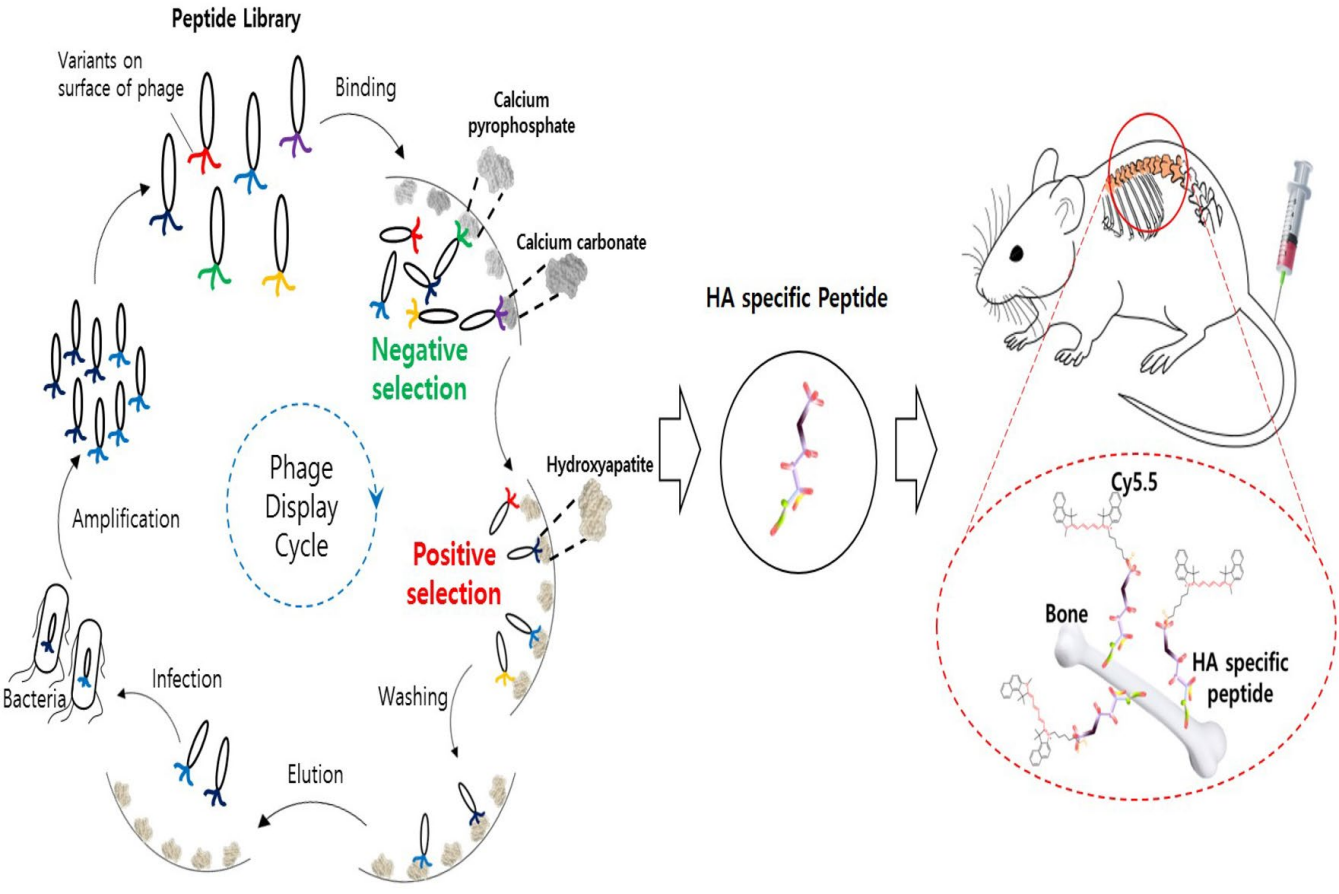
Overview of the screening of hydroxyapatite specific peptides and in vivo bone-targeting imaging.

To isolate highly specific peptides to hydroxyapatite, we used negative selection, which can remove weakly or nonspecifically binding peptides from the phage library (Fig. 1). The randomized 8-mer peptide library was pre-incubated in other calcium-based materials, such as calcium carbonate or calcium phosphate, to eliminate nonspecifically bound phages. We then performed positive phage display selection with hydroxyapatite. After the completion of negative and positive selection, hydroxyapatite specific peptides with fluorescent probes were synthesized and administered to mice by a tail vein injection. Finally, we can see the real-time bone-specific in vivo imaging using a whole-body fluorescence imaging system.

### Phage selection of hydroxyapatite-binding peptides

**Figure 2:**
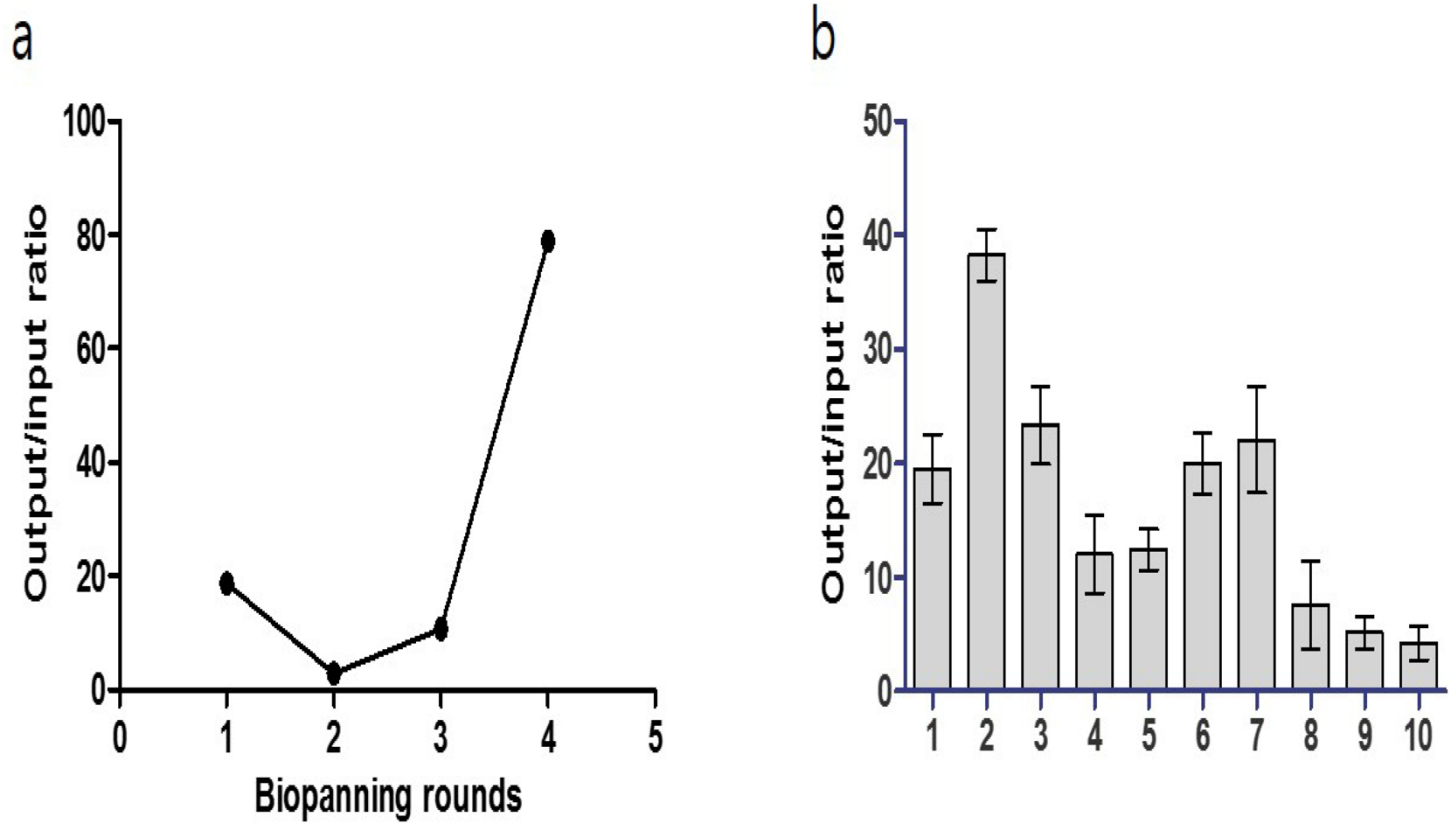
Phage selection of hydroxyapatite binding peptides from a randomized 8-mer peptide phage library. (**a**) Ratio of output/input phages after one, two, three, and four rounds of selection against hydroxyapatite. (**b**) The ratio of the output/input phage of hydroxyapatite-specific positive clones after the fourth biopanning.

We screened hydroxyapatite-specific peptides from a randomized 8-mer peptide phage library, which was constructed by NNK codon-based randomization (N = A or C or G or T; K = G or T). The combinatorial peptide library was composed of 1 × 10^8^ independent peptide clones and was displayed by the N-terminus of the pIII protein of M13 bacteriophage. In vitro phage-display screening with negative and positive selection was used to isolate highly specific peptides that can bind to hydroxyapatite during four rounds of biopanning. To increase the selection stringency, the number of washings was increased from five to ten for each round of selection using 0.05% PBST (Phosphate Buffered Saline with Tween 20). Significant enrichment of hydroxyapatite binders (40-fold increase) was obtained after four rounds of biopanning (Fig. 2a), and at that stage ten peptide phages were selected for binding to hydroxyapatite using the output/input phage ratio (Fig. 2b). DNA sequencing revealed that all 10 clones had unique sequences. Among them, three peptide molecules with output/input phage ratios of more than 20 were selected for further characterization and were labeled HA-pep2, HA-pep3, and HA-pep7. Their sequences are presented in Table 1. The molecular weight of the three peptides with 8 amino acids was almost 1,000 Da and all of them included positive amino acids (Lysine, Arginine, Histidine). Interestingly, all three peptides showed positive net charge with an isoelectric point (pI) of 11.7, 11.0, and 8.6, respectively. We analyzed secondary structure of peptides using GOR (Garnier-Osguthorpe-Robson) protein secondary prediction method. As a result, HA-pep2 consists of 75% random coil and 25% extended strand. HA-pep3 has 62.5% random coil and 37.5% extended strand. HA-pep7 shows 100% random coil structure. Therefore, the binding affinity of peptides is increased according to the increase of extended strand secondary structure.

**Table 1:** Summary of representative binding peptides (HA-pep2, HA-pep3, and HA-pep7) to hydroxyapatite.

Number of functional amino acid residues							
Peptide	Sequence	Basic	Alcohols	Aliphatic	Aromatic	PI	Secondary structure
HA-pep2		4	1	2	0	11.7	ccccceec
HA-pep3		3	2	0	1	11.0	cccceeec
HA-pep7		1	2	1	2	8.6	cccccccc

Blue = basic, red = alcohols, green = aliphatic, orange = aromatic, black = all others, c = random coil, e = extended strand.

### Binding/release studies of HA-binding peptides

**Figure 3:**
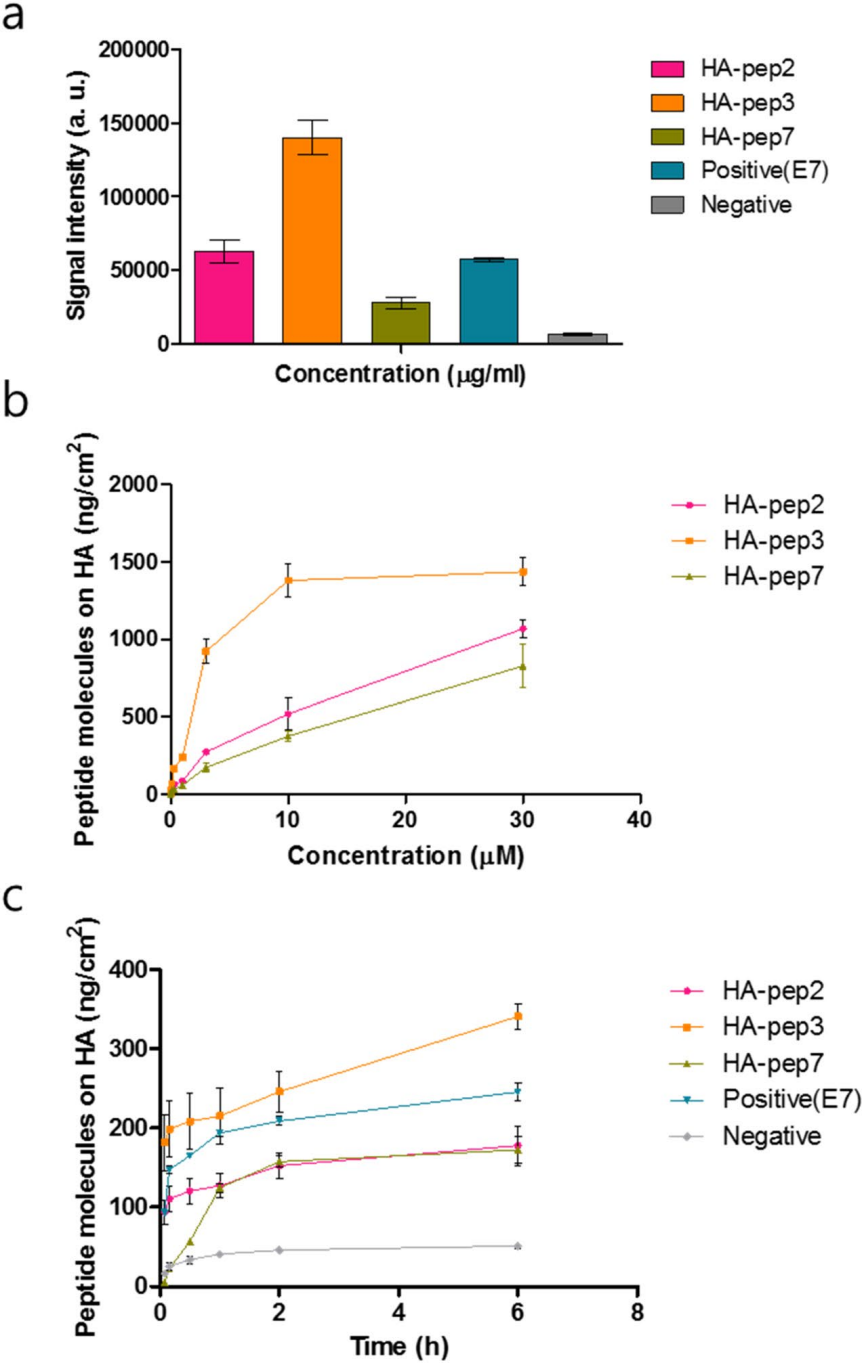
Binding studies of FITC-labeled HA-specific peptides. (**a**) HA-binding test of selected peptides (HA-pep2, HA-pep3, HA-pep7), positive peptide(E7) and negative peptide. (**b**) Binding assay of HA-binding peptides in different concentrations and (**c**) incubation times.

We analyzed the fluorescent signal of FITC-labeled peptide solutions before and after incubation with hydroxyapatite, to quantify the binding ability of the three selected peptides to hydroxyapatite (Fig. 3a). At dose 3 μg/mL, HA-pep3 showed the highest fluorescent signal (HA-pep2: 63,153.5, HA-pep3: 140,010.3, HA-pep7: 28,020.6, positive peptide(E7): 57,292 and Negative: 6,592).

To investigate the concentration-dependent effect of the peptides, we measured the fluorescent signal after incubation with hydroxyapatite of 0.01, 0.03, 0.1, 0.3, 1, 3, 10 and 30 μM of the three peptides (Fig. 3b). HA-pep3 clearly had higher HA-binding affinity in comparison with the other peptides and reached saturation above 10 μM. For binding affinity, hydroxyapatite was interacted with FITC-labeled HA-pep3 and positive peptide (E7) at different concentrations (0.01, 0.03, 0.1, 0.3, 1, 3, 10 and 30 μM in PBS) for 1 h. As shown in Fig. 3b and Figure S1, the HA-pep3 and positive peptide (E7) have a dissociation constant (*K*_*d*_) of ~ 5 μM and ~ 15 μM, respectively.

We investigated binding activity at 5 min, 10 min, 30 min, 1 h, 2 h, and 6 h, to assess the effect of incubation time prolongation. HA-pep2, HA-pep3, and positive peptide (E7) began to fast binding and each binding amount was 93.7 ng/cm^2^, 181.7 ng/cm^2^, 92.96 ng/cm^2^ at 5 min. However, HA-pep7 showed slow binding and the binding amount was 56.42 ng/cm^2^ at 5 min. In additions, the amount of HA-pep2, HA-pep3, HA-pep7, and positive peptide (E7) increased with the incubation time prolongation and had 177.8 ng/cm^2^, 340.9 ng/cm^2^, 172.3 ng/cm^2^, and 245.4 ng/cm^2^ peptide molecules on HA after 6 h, respectively (Fig. 3c).

**Figure 4:**
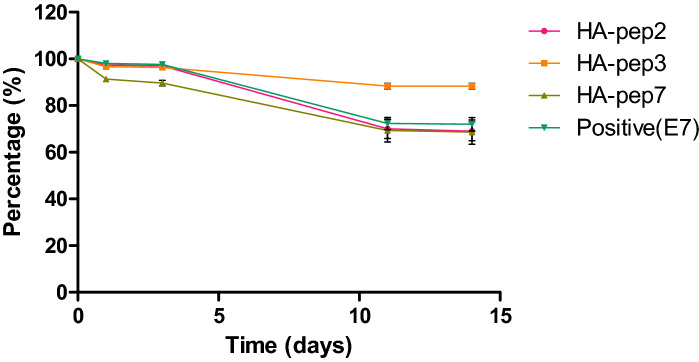
Release kinetics of HA-binding peptides and positive peptide (E7) on hydroxyapatite.

To identify the release kinetics of the three peptides, we performed release tests for 14 days under 37 °C (Fig. 4). HA-pep3 exhibited the lowest burst release at 11.7% of the total binding amount, and it preserved over 88.3% of the immobilized molecules after two weeks. HA-pep2, HA-pep7 and positive peptide (E7) exhibited similar release bursts at 31% ,31.4% and 28% of the total binding amounts. HA-pep3 exhibited better release kinetics toward hydroxyapatite over time (~ 88.3% retained) than positive peptide E7 (~ 72% retained)^16^. Thus, considering the binding ability and release kinetics, HA-pep3 is the most promising peptide for use as a targeting ligand.

### Specificity of HA-pep3

The specificity of HA-pep3 and acidic oligopeptide (poly-Glu amino acids), an established HA-binding peptide, were measured specificity assay in the same molar mass (mass/mol wt) of the biologically relevant calcium salts hydroxyapatite (HA, Ca_5_(PO_4_)_3_(OH)), calcium carbonate (CC, CaCO_3_), calcium phosphate (CP, Ca_3_(PO_4_)_2_), and calcium pyrophosphate (CPP, Ca_2_P_2_O_7_). FITC-labeled HA-pep3 and acidic oligopeptide were incubated for 3 h in various calcium salts and were thoroughly washed using 0.05% PBST (Phosphate Buffered Saline with Tween 20). We then visualized bound peptides utilizing a fluorescence imaging system and measured the fluorescent signal quantitatively.

**Figure 5:**
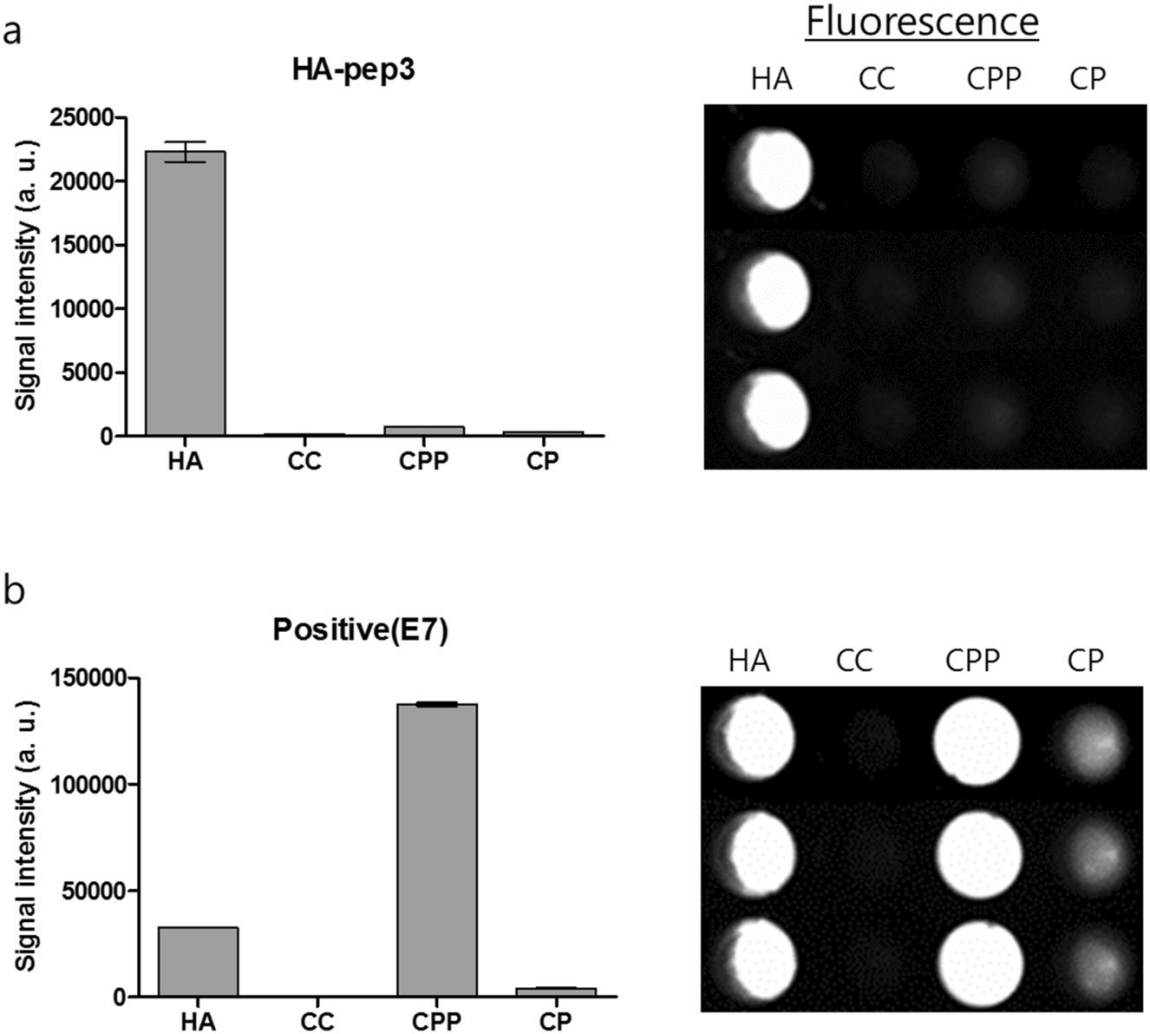
The specificity of (**a**) the HA-pep3 and (**b**) acidic oligopeptide as positive peptide for the biologically relevant calcium salts. HA = hydroxyapatite, CC = calcium carbonate, CPP = calcium pyrophosphate, CP = calcium phosphate.

As shown in Fig. 5a, HA-pep3 demonstrated stronger affinity and higher specificity for hydroxyapatite compared with other calcium salts. There was very low adhesion of HA-pep3 to calcium carbonate, implying that HA-pep3 is not selectively interacting with calcium only. Importantly, we also observed that HA-pep3 did not bind to calcium phosphate, indicating that HA-pep3 was not recognizing the phosphate group only of the mineral. Indeed, the selective hydroxyapatite-binding of HA-pep3 demonstrated that the HA-pep3 interaction is dependent on both the chemical composition of the mineral and the defined physical arrangement of these components on the surface. We performed a competitive binding study with 10-, 50-fold excess unlabeled HA-pep3 and FITC-labeled HA-pep3 in hydroxyapatite. As shown in Figure S2, the signal of FITC-labeled HA-pep3 with 50-fold excess unlabeled HA-pep3 was reduced from 24,956 to 8,130 (67% reduction). As a result, we demonstrated that HA-pep3 is specific for hydroxyapatite.

However, the acidic oligopeptide(E7) had no specificity for hydroxyapatite; it showed a higher binding signal on calcium pyrophosphate than on hydroxyapatite (Fig. 5b). Therefore, HA-pep3 enables the fluorescence detection of hydroxyapatite as a bone-targeting probe with high sensitivity unlike acidic oligopeptide(E7).

### In vivo bone-targeting imaging of HA-pep3

**Figure 6:**
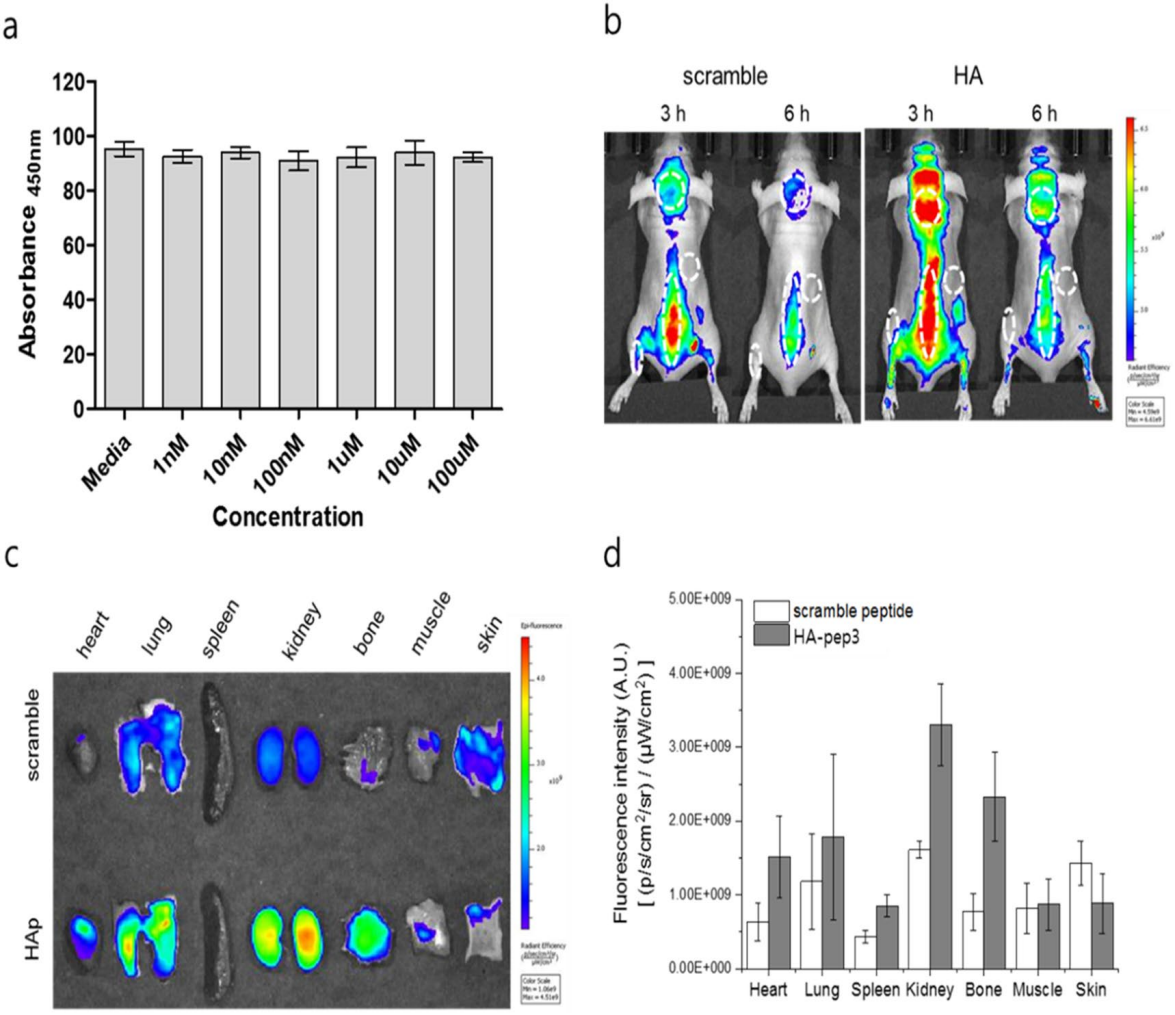
In vivo application of the HA-pep3. (**a**) Viability of Saos-2 cells treated with HA-pep3. (**b**) In vivo fluorescence imaging of BALB/c nude mice at 3 and 6 h after intravenous injection of Cy5.5-scramble peptide and Cy5.5-HA pep3. Skin, skull, spine, and joint are marked as dashed lines. (**c**) Ex vivo fluorescence imaging of bone and major tissues using the HA-pep3 and scramble peptide. (**d**) Fluorescence signal intensity graph of HA-pep3 and scramble peptide in bone and major tissues.

The biocompatibility of HA-pep3 is important for assessing the feasibility of its in vivo application. Therefore, we performed HA-pep3 cytotoxicity assessment in osteoblastic Saos-2 osteogenic cells for 24 h, 48 h and 72 h (Fig. 6a and Figure S3). The cell viability was measured using various concentrations of HA-pep3 ranging from 1 nM to 100 μM. HA-pep3 exhibited no cytotoxicity even at high concentrations (100 μM). Next, we analyzed HA-pep3 in vivo application feasibility. We used Cy5.5 dye with near-infrared wavelength for in vivo imaging, to minimize tissue adsorption^20^. After Cy5.5 dye labeling, scrambled peptide and HA-pep3 were injected into the tail vein of nude mice. During real-time whole-body imaging, the accumulation of Cy5.5-HA-pep3 in the skull, spine, and joints was higher than that exhibited by a scrambled control peptide (Fig. 6b) at both 3 and 6 h after injection. Ex vivo analysis of the major mice organs and bone showed similar trends with bone-targeted binding of Cy5.5-HA-pep3 (Fig. 6c). The fluorescence intensity in bone was about 3.32 folds higher for Cy5.5-HA-pep3 than for a scrambled sequence (Fig. 6d). We also observed high accumulation of Cy5.5-HA pep3 in spine after intravenous injection to wild type C3H/HeN mice (Figure S4).

We used X-ray function of in vivo imaging machine, IVIS Lumina XRMS and performed X-ray imaging of the mice with Cy5.5-HA pep3. With x-ray images, we could observe the high fluorescence intensity at spine (Figure S5a). In additions, we compared its biodistribution in vivo to our Cy5.5-HA pep3 and E7 positive peptide after intravenous injection. In Figure S5, our HA pep3 showed superior bone targeting and accumulation compared to the known calcium binding and HA binding peptide (E7 peptide). In addition, we performed blood analysis to determine clearance of our peptide in vivo (Figure S6). Most of HA pep3 disappeared in blood after 12 h showing its short circulation time which is advantageous for minimizing background signals. These data demonstrate the potential of HA-pep3 for successful use as a bone-targeting probe for in vivo imaging.

## Discussion

It is well established that peptide targeting ligands are advantageous in achieving high signal to noise due to rapid renal excretion and in being very specific to the target. Furthermore, they exhibit no apparent adverse effects, such as immunogenicity and cytotoxicity. In nature, acidic peptide sequences derived from bone matrix proteins, osteopontin, and bone sialoprotein, are known to bind strongly to bone mineral surfaces^21–23^. The repeating units of Glu or Asp have high affinity to hydroxyapatite, which is implicated in the coordination of calcium ions in a hydroxyapatite crystal lattice^24^. Due to this property, the use of acidic oligopeptides in various biomedical applications, such as HA powder^25,26^, discs^27^, HA-Ti implants^28^. and bone allografts^29^, has been reported. Thus, acidic oligopeptides conjugated to fluorescent probes were utilized to evaluate their affinity for HA both in vitro and in vivo^15^. The in vivo analysis in mice showed that probes with six or more acidic repeat sequences were accumulated into the bone. In addition, acidic oligopeptide conjugated drug delivery carriers were developed for bone targeted therapy^30,31^. The use of acidic oligopeptides for bone imaging is an attractive option, because they exhibit no apparent side-effects and have a shorter half-life in vivo compared to bisphosphonates.

However, our study demonstrated that acidic oligopeptide has poor specificity for several calcium salts-based materials. For bone targeting and imaging, peptide ligands with improved specificity are required. Therefore, we screened novel peptides with strong affinity and high specificity for binding hydroxyapatite from a randomized 8-mer peptide phage library using the methods of negative and positive selection. Hydroxyapatite-binding peptides screened from phage display or combinatorial peptide libraries have been reported previously^32–34^. However, most of these peptides have been used to control the nucleation and mineralization of hydroxyapatite formation. To the best of our knowledge, this is the first report describing the isolation of novel hydroxyapatite-binding peptides and their use for bone-specific in vivo imaging.

After phage-display selection, the peptides HA-pep2, HA-pep3, and HA-pep7 were identified as having strong and specific affinity to hydroxyapatite. Our data show that HA-pep3 has the best HA-binding properties among the three isolated peptides. Compared to acidic oligopeptide (E7) as a positive control, HA-pep3 demonstrated higher binding ability to hydroxyapatite (HA-pep3: *K*_*d*_ = 5 μM vs positive peptide (E7): *K*_*d*_ = 15 μM). In addition, HA-pep3 had a higher dissociation constant toward hydroxyapatite surfaces over time (~ 88.3% retained) than the one reported for acidic oligopeptide (~ 72% retained). Moreover, HA-pep3 is highly specific for hydroxyapatite compared to other calcium salt based materials, such as calcium carbonate, calcium phosphate, and calcium pyrophosphate, whereas acidic oligopeptide is nonspecific.

Surprisingly, amino acids such as Glu(E) and Asp(D), known for their binding affinity to hydroxyapatite, are not present in HA-pep3 (KNFQSRSH), and the net charge of the peptide is highly positive (pI = 11.7). The binding mechanism between HA-pep3 and hydroxyapatite requires future investigation. HA-pep3 with positive net charge did not show any cytotoxicity up to 100 μM compared to cytotoxicity at 10 μM for the poly-arginine oligopeptide with positive net charge^35^. HA-pep3 is safer, because it is composed of positive charge amino acid, hydrophobic and hydrophilic amino acids. These properties represent an attractive feature for in vivo targeting and imaging utilization.

As an in vivo application, we labeled the HA-pep3 with Cy5.5 dye and injected it into the tail vein of mice. Biodistribution of the injected materials is determined by various factors including size, charge, and hydrophobicity^36^. For successful in vivo imaging, the materials need to provide sufficient contact time with the target, fast and strong binding to the target, and minimization of non-specific binding to other tissues. Furthermore, the Choi group has shown that the chemical structure of labeled dye molecules plays an important role in determining the in vivo fate of materials^37,38^. In this study, we used the commercially available Cy5.5 dye for labeling. Therefore, we expect that rational design of the resulting conjugate and selection or synthesis of novel dye molecules will further improve the bone-targeting ability of HA-pep3 in vivo.

## Conclusions

In this study, we identified a peptide exhibiting high specificity binding to hydroxyapatite, a major inorganic component of teeth and bones, using negative and positive selection approaches from a randomized 8-mer peptide phage library. Unlike acidic oligopeptide, a well-known HA-binding peptide, HA-pep3 showed high specificity for hydroxyapatite in comparison with other calcium salts, highlighting the potential of HA-pep3 to serve as a bone-targeting probe for in vivo imaging. Our newly developed peptide is useful to bone imaging as well as a drug delivery system by conjugating this peptide on a drug carrier as a targeting ligand. We expect that efforts for the development of novel bone targeting peptides will open new avenues for improved targeted imaging of bones, which will potentially benefit the diagnosis and treatment of skeletal diseases such as osteoporosis.

## Methods

### Ethics statement

The Animal Care Committee of Catholic University of Korea approved the animal experimental protocols. All animal experiments were conducted in accordance with the protocols approved by the Animal Research Ethics Committee at the Catholic University of Korea (Approval No. CUMC-2019-0003-01). All experimental procedures performed followed the ethical guidelines on animal use.

### Materials

Phosphate-buffered saline (PBS), Tween20, polyethylene glycol (weight-averaged molecular weight (*M*_*W*_) = 8,000 g mol^−1^), LB agar, hydroxyapatite, calcium carbonate, calcium pyrophosphate, calcium phosphate salts, and bovine serum albumin (BSA) were purchased from Sigma-Aldrich (St. Louis, MO, USA). All peptides used in this study were obtained from Anygen (South Korea). Normal saline (0.9%) was purchased from Daihan pharm. Co., Ltd. (Seoul, Yeongdeungpo-gu, Korea).

### Phage selection of HA-specific peptides

An 8-mer peptide phage library was constructed by NNK codon-based randomization (N = A or C or G or T; K = G or T). The randomized gene fragments (NNK)_8_ were double-digested with SfiI/NotI (New England Biolabs) and cloned into pIGT2 phagemid vectors (IgTherapy Co.). The cloned vectors were transformed into *E. coli ER* cells; the library was composed of 1 × 10^8^ independent peptide clones. The peptide recombinant phage library was prepared using Ex 12 helper phage (Ig Therapy Co.), which is displayed by the N-terminus of the pIII protein of M13 bacteriophage.

Hydroxyapatite (HA) (2–3 mm wide and 2 mm thick) was used as the target during phage selection. Hydroxyapatite was incubated in blocking buffer (PBS containing 2% BSA) for 2 h at room temperature. Calcium carbonate and calcium phosphate salts, utilized for negative selection, were also incubated in blocking buffer. First, the prepared peptide recombinant phages (1 × 10^11^ plaque-forming units [PFU]) were added to calcium carbonate and calcium phosphate for negative selection for 1 h at 30 °C to remove nonspecific phages. Then, unbound phages were incubated in hydroxyapatite for positive selection for 1 h at 30 °C and were washed with PBS containing 0.05% Tween20 (five times since then round ten times). Bound phages eluted by incubation with 0.2 M glycine–HCl (pH 2.0) for 20 min, followed by immediate neutralization with 1 M Tris (pH 9.0). For the next biopanning, the eluted phages were infected into *E. coli ER* (Stragagene) for 1 h at 37 °C, and helper phages (5 × 10^9^ PFU) were added and prepared next recombinant phage library. At each step, the output/input phage ratio in all rounds of biopanning were measured. After the fourth biopanning, twenty clones with the highest output/input phage ratio were randomly selected. The clones were analyzed to DNA sequencing using a phagemid primer (5′-GQTTACGCCAAGCTTTGGAGC-3′; Bioneer).

### Characterization of HA-binding peptides

FITC-labeled peptides HA-pep2, HA-pep3, and HA-pep7 were synthesized (Anygen). For binding tests, hydroxyapatite was interacted with FITC-labeled peptides at different concentrations (0.01, 0.03, 0.1, 0.3, 1, 3, 10 and 30 μM in PBS) for 1 h. The hydroxyapatite was also incubated in 2 μg/mL FITC-labeled peptide PBS solution for different time periods (5 min, 10 min, 30 min, 1, 2, and 6 h). Release tests were carried out on the hydroxyapatite after incubation in 2 μg/mL FITC-peptide PBS solution for 14 days. The release test was performed in incubation at 37 °C. All tests were carried out in triplicates, and original hydroxyapatite was used as negative control. The presence and quantification of the peptide on the HA surface were assessed by measuring the residual fluorescence of the solution (excitation: 488 nm; emission 526 nm) using a Gemini EM fluorescence microplate reader (Molecular Devices, Sunnyvale, CA).

### Calcium salts specificity experiments

Hydroxyapatite (HA, 502.31 g/mol), calcium carbonate (CC, 100.08 g/mol), calcium pyrophosphate (CPP, 254.053 g/mol), and calcium phosphate (CP, 310.2 g/mol) salts were incubated with HA-pep3 and with acidic oligopeptide as positive control (2 μg/mL) in PBS at room temperature for 3 h. Calcium salts were washed three times with 0.05% PBST (Phosphate Buffered Saline with Tween 20) by centrifugation, transferred to a 96-well black plate, and assessed with a fluorescence imaging system. All fluorescence images were collected at identical exposure times and are displayed with equal normalization.

### Cytotoxicity assay of HA-pep3

Saos-2 osteosarcoma osteogenic primary cell line was purchased from the Korean cell line bank. Cells were cultured in RPMI1620 medium with L-glutamine (300 mg/L), 25 mM HEPES, and 25 mM NaHCO3 supplemented with 10% heat-inactivated fetal bovine serum (FBS), 100 U/mL penicillin, and 100 μg/mL streptomycin. The cell lines were cultured at 37 °C and up to 5% CO_2_ in humidified atmosphere. All reagents and cell culture media were purchased from the WELGENE Company, South Korea. HA-pep3 cytotoxicity was examined using WST-1 assay. The peptide was dissolved in deionized water and further diluted in RPMI medium to prepare working concentrations of 1, 10, 100, 1,000, 10,000, and 100,000 nM. The cells were cultured in 96-well plates at a density of 2 × 10^4^ cells per well. After 24 h incubation, the cells were treated with different sample concentrations and incubated for further 24 h. Then, 50 μL WST-1 solution was added to each well, and the plate was re-incubated for 4 h. Finally, the absorbance was measured at 450 nm using a microplate spectrophotometer (Tecan Infinite 200). All assays were carried out in triplicates.

### In vivo and ex vivo imaging

All animal studies were approved by the Institutional Review Board of the Catholic University of Korea (approval No. CUMC-2019-0003-01). BALB/c nude mice (4 weeks old, OrientBio, Seongnam city, Korea) and wild type C3H/HeN mice were used for in vivo imaging. Cy5.5-scramble peptide or Cy5.5-HAp peptide (5 mg/kg of peptide in 100 µL physiological saline, n = 3) were administered to the mice by a tail vein injection. Subsequently, mice were anesthetized by isoflurane inhalation, and whole-body imaging was performed with an IVIS Lumina XRMS (PerkinElmer Inc., Waltham, Massachusetts, USA) set at 660/710 nm at 3 and 6 h post-injection. All images were analyzed with Living Image 4.5 software (PerkinElmer Inc., Massachusetts, USA). Three hours post-injection, bone, muscle, skin, and major organs (heart, lung, spleen, and kidney) were dissected and imaged similarly using IVIS Lumina XRMS. X-ray images of the mice were obtained simultaneously by same machine. As control, E7 peptide was also labeled and teste in vivo similarly. We collected blood samples of 10 μL from mice at different time points (1, 3, 6, and 12 h) after intravenous injection of Cy5.5-HA pep3. The fluorescence intensity of Ce6 in samples was measured by IVIS Lumina XRMS.

## Supplementary information

Supplementary information
