# Supplementary material for: Selection and identification of a novel bone-targeting peptide for biomedical imaging of bone
Source: Sci Rep. 2020 Jun 29;10:10576. doi: 10.1038/s41598-020-67522-4 (PMC7324617; doi:10.1038/s41598-020-67522-4)
Supplement: Supplementary file 1 — Supplementary information [file 41598_2020_67522_MOESM1_ESM.docx]

**Supplementary Information**

**Selection and identification of a novel bone-targeting peptide for biomedical imaging of bone**

Jinho Bang ^a, +^, Heesun Park^a,c, +^, Jihye Yoo ^b^, Donghyun Lee^b^, Won Il Choi ^a^, Jin Hyung Lee ^a^, Young-Ran Lee ^a^, Chungho Kim *^c^*, Heebeom Koo^b, *^, Sunghyun Kim ^a, *^

^a^ Korea Institute of Ceramic Engineering and Technology, Center for Convergence Bioceramic Materials, 202 Osongsaengmyeong 1-ro, Cheongjusi, Chungcheongbuk-do 28160, South Korea

^b^Department of Medical Life Sciences, College of Medicine, The Catholic University of Korea 222 Banpo-daero, Seocho-gu, Seoul 06591, South Korea

*^c^Department of Life Sciences, Korea University, Seoul 136-701, South Korea*

* To whom the correspondence should be made

E-mail: [shkim0519@kicet.re.kr](mailto:shkim0519@kicet.re.kr) / Tel: +82-43-913-1512

E-mail: hbkoo@catholic.ac.kr / Tel: +82-2-2258-7231

Figure S1. Binding test of positive and negative peptides in different concentration

**
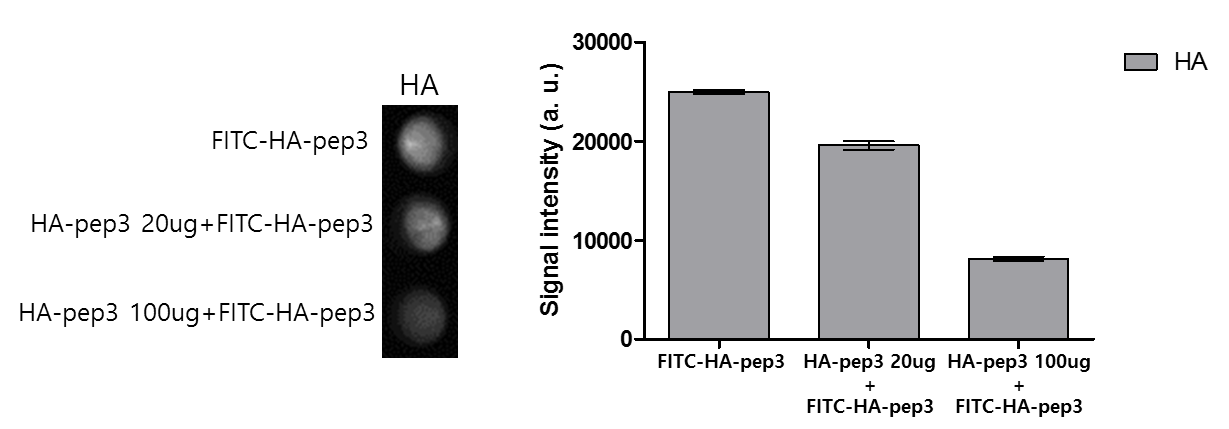
**

Figure S2. Competitive binding study with unlabeled HA-pep3 and FITC-labeled HA-pep3 for specificity.

Figure S3. Viability of Saos-2 cells treated with HA-pep3 after 48 h and 72 h.


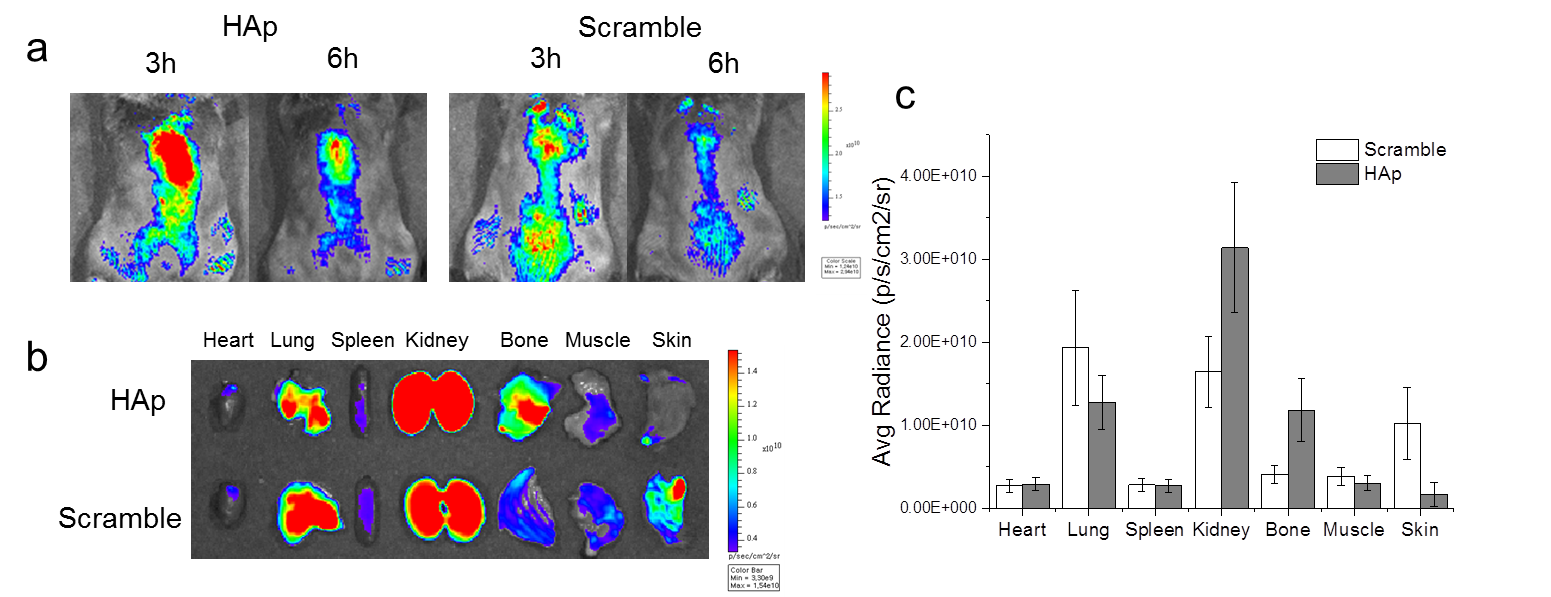


Figure S4. *In vivo* imaging of HA pep3 in wild mice. (a) *In vivo* fluorescence images of C3H/HeN mice at 3 and 6h after intravenous injection of Cy5.5-scramble peptide and Cy5.5-HA pep3. (b) *Ex vivo* fluorescence images of bone and major tissues using the HA-pep3 and scramble peptide. (c) Average fluorescence intensity in (b).

In addition, we performed the prediction of immunogenicity using program of predicted antigenic peptides (http://imed.med.ucm.es/Tools/antigenic.pl). For example, bovine serum albumin (BSA) were predicted that there are 30 antigenic determinants. However, in case of HA-pep3, there is 0 antigenic determinants.


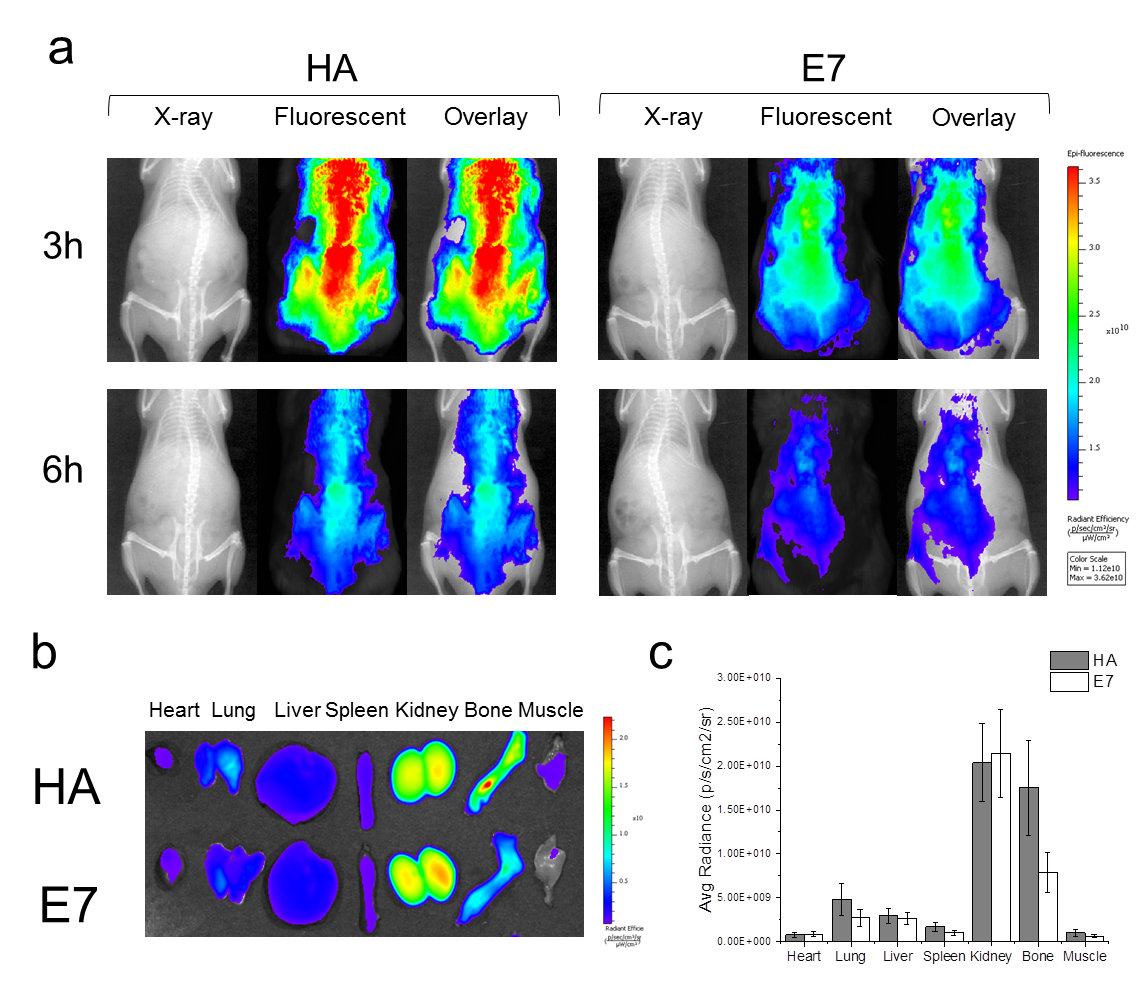


Figure S5. *In vivo* distribution of the NIRF dye labeled HA-pep3 and E7 peptide. (a) *In vivo* time-dependent whole body fluorescence and x-ray images. HA-pep3 and E7 with equal fluorescence intensity were intravenously injected into the mice. (b) *Ex vivo* fluorescence images of bone and major organ using the HA-pep3 and E7 peptide. (c) Average fluorescence intensity in (b).


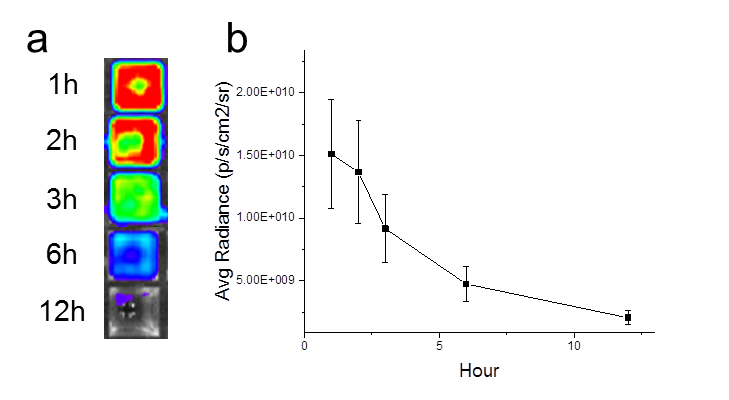


Figure S6. In vivo clearance of HA pep3 from blood. (a) Time-dependent fluorescence images of blood sample obtained from mice tail at 1, 2, 3, 6 and 12h after intravenous injection of Cy5.5-HA pep3. (b) Average fluorescence intensity graph of the blood samples in (a).
